# The health potential of neighborhoods: A population-wide study in the Netherlands

**DOI:** 10.1016/j.ssmph.2021.100867

**Published:** 2021-07-07

**Authors:** L.H. Dekker, R.H. Rijnks, J.O. Mierau

**Affiliations:** a University Medical Center Groningen, Department of Nephrology, Hanzeplein 1, 9713, GZ, Groningen, the Netherlands; b Aletta Jacobs School of Public Health, Landleven 1, 9747, AD, Groningen, the Netherlands; c University College Cork, Cork University Business School, West Wing, Main Quadrangle, T12 K8AF, Ireland; d University of Groningen, Faculty of Economics and Business, Nettelbosje 2, 9747, AE, Groningen, the Netherlands

**Keywords:** Population health, Self-assessed health, Chronic disease, Neighborhoods, Spatial epidemiology, Spatial spillover effects

## Abstract

**Background:**

While differences in population health across neighborhoods with different socioeconomic characteristics are well documented, health disparities across neighborhoods with similar socioeconomic characteristics are less well understood. We aimed to estimate population health inequalities, both within and between neighborhoods with similar socioeconomic status, and assessed the association of neighborhood characteristics and socioeconomic spillover effects from adjacent neighborhoods.

**Methods:**

Based on Dutch whole-population data we determined the percentage of inhabitants with good or very good self-assessed health (SAH) and the percentage of inhabitants with at least one chronic disease (CD) in 11,504 neighborhoods. Neighborhoods were classified by quintiles of a composite neighborhoods socioeconomic status score (NSES). A set of spatial models was estimated accounting for spatial effects in the dependent, independent, and error components of the model.

**Results:**

Substantial population health disparities in SAH and CD both within and between neighborhoods NSES quintiles were observed, with the largest SAH variance in the lowest NSES group. Neighborhoods adjacent to higher SES neighborhoods showed a higher SAH and a lower prevalence of CD. Projected impacts from the spatial regressions indicate how modest changes in NSES among the lowest socioeconomic neighborhoods can contribute to population health in both low- and high-SES neighborhoods.

**Conclusion:**

Population health differs substantially among neighborhoods with similar socioeconomic characteristics, which can partially be explained by a spatial socio-economic spillover effect.

## Highlights

• Variations in health within places with similar socio-economic status (SES) are understudied.
• Policy makers have increasing interest in unravelling the determinants of such variation.
• Substantial health disparities among neighborhoods with similar SES exist.
• The variation is partially the result of spatial spillover effects.
• Neighborhoods adjacent to higher SES neighborhoods revealed better population health.

## Background

A large body of literature documents a socioeconomic gradient in population health at the neighborhood level (Duncan & Kawachi, 2018). More affluent neighborhoods exhibit better health outcomes such as a lower risk of stroke (Howard et al., 2016), diabetes (Bilal et al., 2018; Corriere et al., 2014), asthma (Kim et al., 2018), and higher values of a variety of quality-of-life measures (Rocha et al., 2017). Furthermore, low neighborhood socioeconomic status (NSES) is associated with relatively high healthcare costs (de Boer et al., 2019), due to, amongst other factors, more hospital admissions (Asaria et al., 2016). While the positive socioeconomic gradient (i.e., higher incomes associated with better health) is commonly established, it is not ubiquitous. Diabetes, for instance, displays an inverse socioeconomic gradient in many low- and middle-income countries, while displaying a positive gradient in many high-income countries (Seiglie et al., 2020). Similarly, focusing on the Netherlands, de Boer et al. (2020) find that, overall, lower NSES is associated with worse health behavior but that this is notably different for excessive alcohol use, which is particularly prevalent among high-income neighborhoods.

While differences in population health across neighborhoods with different socioeconomic characteristics are well documented, health disparities - and their causes - across neighborhoods with similar socioeconomic characteristics are less well understood. Ferrer and Palmer (2004) speculate that there may be an interaction between NSES and individual characteristics, where the effect of NSES on health is exacerbated when individual characteristics are unfavorable. This speculation is corroborated by Zhu et al. (2021) who show that individuals with individual disadvantage have worse lifestyles in neighborhoods that are disadvantaged than in neighborhoods that are not. In addition, recently, lifestyle has been found to act as a potential individual determinant that causes variation in health within neighborhoods with similar socioeconomic characteristics (de Boer et al., 2020). Additionally, contextual neighborhood determinants may also play a role. For example, population density has been found to largely account for cardiovascular health disparities between metropolitan and smaller community areas (Lawrence et al., 2017).

While there is a vast literature on the interplay between health and place, commensurately less is known about the relationship between the spillover effects of the social, demographic, geographic and economic characteristics of one neighborhood toward its adjacent neighborhoods. It may be hypothesized, however, that similar mechanisms are at play and that, for instance, the availability of facilities in one neighborhood also affect the well-being of residents of adjacent neighborhoods. Spillovers may occur when there is social interaction and movements among individuals located in a close spatial proximity which extends beyond the arbitrary defined neighborhood boundaries (Browning & Soller, 2014; Duncan & Kawachi, 2018). Along those lines, being adjacent to a high SES neighborhood which are known to have better facilities, can have a positive effect on the neighboring neighborhood even if that neighborhood itself is, in fact, a low SES neighborhood.

The vast majority of existing studies (Chaix, 2009; Diez Roux, 2008; Mair et al., 2008; Meijer et al., 2012) do not account for the potential of spatial relationships between geographic locations in their multilevel regression analyses (Oka & Wong, 2016). Therefore, Diez Roux (2008), Spielman and Yoo (2009) and Perchoux et al. (2013) have criticized that the neighborhood characteristics surrounding the residents are not adequately represented and analyzed in the current literature. This is unfortunate as there is a strong basis to suggest that “where we live” matters for our health in addition to “who we are” (Duncan & Kawachi, 2018). Nevertheless, in literature there is a emerging focus on relative deprivation between adjacent neighborhoods. Cox et al. (2007), for instance, highlight that while Type 2 Diabetes is more prevalent in deprived neighborhoods, deprived neighborhoods that are surrounded by less deprived neighborhoods have a lower prevalence. Zhang et al. (2011) show that deprivation but also relative deprivation (i.e., living in a neighborhood that is more deprived than its adjacent neighborhoods) is associated with higher mortality rates. Somewhat differently, Allender et al. (2012) found that poor neighborhoods surrounded by rich neighborhoods have worse health than poor neighborhoods surrounded by poor areas. As such, the existence of spatial spillover seems clear, albeit that the direction is not always the same.

Against the above backdrop, we aimed to estimate population health inequalities, both within and between neighborhoods with similar socio-economic status, and assessed the association of neighborhood characteristics and socio-economic spillover effects from adjacent neighborhoods.

## Methods

### Data

We used population health and lifestyle measures from the Dutch Public Health Monitor 2016 of the National Institute for Public Health (RIVM). The Dutch Public Health Monitor is held every four years and is based on a national survey of over 400,000 inhabitants, containing data on self-reported health, health perception, and health-related behaviors of persons aged 19 years and older. Using structured additive regressions, the survey data is translated into valid small area estimates that can potentially be used for health policy decisions (van de Kassteele et al., 2017). The small areas for which the RIVM provides data are the Statistics Netherlands neighborhoods, which are the smallest administrative regions defined by Statistics Netherlands. Neighborhoods in this study range in size from 50 inhabitants to a maximum of 28,120, with a mean of 1473 and median of 850 (the interquartile range is 275–1990). Neighborhood socioeconomic status (NSES) was derived from the dataset for neighborhoods provided by Statistics Netherlands. The NSES was calculated using Nonlinear Iterative PArtial Least Squares Principal Component Analysis (NIPALS), based on neighborhood income statistics (percentage people in lower two quintiles of national income, percentage people in highest quintile), social welfare reliance (percentage of people on benefits, disability benefits, or unemployment benefits), and housing market characteristics (average estimated housing-price, percentage owner-occupied, percentage council housing). The algorithm accommodates missing values in PCA analysis, allowing for more neighborhoods to be included in the paper. The scores on the first component (factor) were taken as the NSES score and were subsequently categorized into quintiles. The first component loadings (see Table 1) show the calculated NSES score corresponds to neighborhoods with a larger share of high-income households, higher average incomes, higher average property values and higher shares of owner-occupied properties. Subsidized renting, share of low-income households, and the share of the population receiving unemployment benefits load negatively on the first component. Both the share of people receiving disability benefits and the share of people receiving short-term unemployment benefits have smaller, albeit still negative, loadings on the first component. The resultant loadings on the first component are in line with expectations of an indicator of neighborhood socioeconomic status. The second component, shown for illustrative purposes and not used in this study, separates owner-occupied (high) from subsidized renting (low), and loads high on unemployment and average income. The second component broadly corresponds to an urban-rural (low-high) geography. The Statistics Netherlands neighborhood dataset contains 12,822 neighborhoods. Of these, 1318 have no socio-economic data due to low numbers of inhabitants, representing a total of 21,520 inhabitants (0.1% of the total dataset). These neighborhoods were removed from the data. All other neighborhoods were included in the analysis. The NSES data combined with the neighborhood health data is available for 11,504 neighborhoods with an average population of 1473 individuals per neighborhood.

**Table 1:** Component loadings socio-economic status.

	PC1	PC2
**Average Property Value**	0.353	0.147
**Percent Owner Occupied**	0.344	-0.485
**Percent Subsidized Rent**	-0.365	0.470
**Percent Low-Income Households**	-0.357	-0.184
**Percent High-Income Households**	0.406	0.318
**Average Income per Recipient**	0.379	0.424
**Percent Unemployment Benefits**	-0.334	0.336
**Percent Disability Benefits**	-0.225	-0.282
**Percent Short Term Unemployment Benefits**	-0.163	0.126

### Dependent variables

We used two health indicators from the Dutch Public Health Monitor data, the percentage of individuals who indicated to be in good or very good health, from now on “self-assessed health” (SAH) and the percentage of individuals suffering from one or more long standing (> 6 months) illnesses or health problems from now on “chronic diseases” (CD). Self-assessed health was assessed on a five point Likert scale. The prevalence of chronic diseases was assessed with a yes/no two-item response. To contextualize our results, the following four lifestyle indicators are also shown in Table 1, all measured at the neighborhood level: 1) the percentage of non-smokers; 2) the percentage of those that adhere to the 2016 Dutch alcohol recommendation; 3) the percentage of individuals who complied with the 2016 Healthy Exercise Guideline; 4) the percentage of individuals with overweight (body mass index ≥ 25 kg/m^2^).

### Explanatory contextual variables

Three potential explanatory contextual factors were assessed. The first two variables were 1) the percentage of inhabitants over the age of 65 years, and 2) the population density of the neighborhood, both provided by Statistics Netherlands at the neighborhood level. As a third explanatory variable we calculated a spatially lagged term for NSES to assess spillover effects of adjacent (neighborhood) SES. For the spatial lag we construct a spatial weights matrix consisting of all neighborhoods within a 12.5 km radius (centroid to centroid), resulting in a minimum of one neighbor per neighborhood. Weights were calculated using 1/distance squared, and subsequently row-standardized to account for heterogeneity in the number of neighbors. The average number of neighbors is 201, the least connected region has one neighbor and the most connected region has 635. The median link distance is 8.0 km (interquartile range is 5.1 to 10.4 km).

### Statistical analysis

Health and contextual characteristics of quintiles of NSES scores were analyzed by descriptive statistics (mean and SD). Kernel density plots were derived to visualize the distribution of SAH and CD across quintiles of NSES scores. The amount of within and between quintiles NSES variance in SAH health and CD was calculated using ANOVA. Linear regression models were applied to assess the role of the explanatory contextual variables in the variation of SAH and CD across quintiles of NSES scores. Subsequent to the parsimonious model, population density and the percentage of inhabitants aged over 65 were added (Model 2).

Building on this baseline a-spatial model, we subsequently assess three types of spatial models. A general introduction and outline of the rationale behind spatial econometric models is given by Vega and Elhorst (2015) and summarized below, starting from the overarching general nested spatial model, followed by the link with the OLS model, and the intuition behind the spatial components. This section draws on the works by Vega and Elhorst (2015), Anselin (1995), and Bivand (2002).

A general nested spatial model (GNS), including all the spatial effects, takes the form

Y=ρWY+α+Xβ+WXθ+u,

u=λWu+ε

where the conventional regression terms *Y* (the dependent variable), (a vector of ones for the intercept), *X*β (the independent variables), and ε (the error term), are complemented with their spatial terms. The three spatial terms are, first, ρWY, representing a spatial autoregressive term of the dependent variable, e.g. rising house prices in one region drawing up the house prices in neighboring regions. Second, WXθ are the dependent variables whose effects spill over into neighboring areas, e.g. amenities such as parks and sports accommodations in one region may affect the quality of life in neighboring regions as well. Finally, λWu accounts for spatial structures in the error term, e.g. some unobserved processes or variables, collected in the error term, may display spatial autocorrelation. The W in the models represents a spatial weights matrix, which determines the spatial connections between the observations. As is apparent from the GNS equation, if all the spatial terms or the spatial weights are constrained to 0, the model defaults back to the OLS model. If the spatial econometric modelling returns insignificant coefficients for either ρ, *θ*, or *λ*, these can be excluded from the parsimonious model.

Conventionally, spatial econometric modelling is performed stepwise from the GNS, incorporating all three effects, and subsequently estimating models with insignificant spatial effects eliminated for a total of eight models ending in the conventional OLS model. Vega and Elhorst (2015) propose a different starting point, showing that the SLX model is more flexible in its ability to measure spillover effects. In addition, they argue that without a strong theoretical model pointing towards a spatially autocorrelated dependent term this model is hard to justify. We follow the structure provided by Vega and Elhorst (2015) and first estimate an SLX model, or spatial lag of the independent variables (Model 3) and a Spatial Durbin Error model (Model 4), which eliminates the spatial lag of the dependent variable from the GNS. We complement these models (Supplementary Tables S1 and S2) with a general nested spatial model (GNS) and a spatial autoregressive combined model (SAC), which eliminates the spatial lag of the independent variables from the GNS, to maintain a close link with the more conventional modelling of spatial econometric models.

Furthermore, we estimate the effect of a hypothetical policy scenario on SAH or CD. The final model incorporates both direct effects, the association between NSES and SAH or CD, and indirect effects through spatial spillovers. As the usual regression coefficients only reveal the direct effect of NSES, showing the predicted impacts of a change in NSES including both these effects provides more insight into the expected effects. For this scenario we take the lowest NSES quintile and raise the NSES to the lower limit of the second quintile. All analyses were performed in R, for the NIPALS we used the NIPALS R package (Wright, 2018), spatial data manipulations were performed using the spatialreg package (Bivand et al., 2013; Bivand & Piras, 2015).

## Results

### Descriptive statistics

The descriptive statistics reveal a typical stepped relation between NSES and health (Table 2). As a neighborhood moves along the socioeconomic ladder, the share of individuals indicating being in good or very good health increases, while the prevalence of CD decreases. Additionally, as a corroboration, the data shows that a higher NSES score is characterized with better lifestyles, i.e., less overweight, less smoking and participating considerably more in sports activities. Those in the highest NSES quintile are, however, less likely to comply with the Dutch alcohol norm. Albeit less pronounced, higher NSES scores were characterized by lower population density and a smaller share of individuals older than 65 years of age. Kernel density plots revealed that there is overlap between the percentage of SAH and CD across the quintiles of NSES scores (Supplementary Figs. S1a and S1b).

**Table 2:** Descriptive statistics on neighborhoods characteristics by quintiles of neighborhood socioeconomic status.

	Total	NSES Q1	NSES Q2	NSES Q3	NSES Q4	NSES Q5
**Neighborhoods (n)**	11,504	2300	2301	2301	2301	2301
**Self-assessed health (%, SD)**	76.9 (6.6)	68.7 (6.7)	75.4 (4.4)	78.6 (3.9)	80.1 (3.7)	81.7 (4.5)
**Chronic diseases (%, SD)**	33.1 (5.4)	38.7 (5.4)	34.7 (3.8)	31.9 (3.8)	30.59 (4.0)	29.7 (4.5)
**Overweight (%, SD)**	49.9 (5.7)	52.2 (6.8)	51.8 (5.1)	50.2 (4.7)	48.7 (4.5)	46.9 (5.3)
**Non-smokers (%, SD)**	20.2 (5.1)	26.41 (4.8)	21.0 (3.6)	18.8 (3.4)	17.9 (3.3)	16.9 (3.6)
**Sports (%, SD)**	49.4 (8.1)	43.1 (7.7)	47.4 (6.2)	50.3 (6.3)	52.1 (6.7)	54.0 (8.2)
**Alcohol recommendation (%, SD)**	39.2 (7.4)	45.9 (8.5)	39.9 (6.0)	37.4 (5.6)	37.0 (5.6)	36.0 (6.2)
**Neighborhood density (n)**	3169 (4004)	5916 (4722)	3646 (3730)	2521 (3465)	2038 (3296)	1727 (3096)
**Over 65 years (%, SD)**	19.1 (9.2)	19.5 (10.9)	20.2 (8.3)	18.6 (7.4)	17.9 (8.0)	19.1 (10.7)

We find substantial population health disparities in SAH and CD both between neighborhoods with different and neighborhoods with similar socioeconomic characteristics (Supplementary Table S3). When we decompose the variation of SAH, CD and lifestyle measures into within and between quintiles of NSES, the within variation is similar to the between variation.

Linear regression analysis shows that the average SAH in the first quintile is just under ten percentage points lower than in the third quintile (Table 3). Population density is negatively associated with SAH, as is the proportion of individuals aged 65 or older.

**Table 3:** SAH Model estimates.

	Model 1: β	Model 1: t-value	Model 2: β	Model 2: t-value	Model 3: β	Model 3: t-value	Model 4 SPD-E: β	Model 4 SPD-E: t
**(Intercept)**	78.62∗∗∗	788.50	84.41∗∗∗	692.09	84.33∗∗∗	700.20	83.804∗∗∗	564.167
**NSES Q1**	-9.91∗∗∗	-70.26	-8.96∗∗∗	-71.80	-8.27∗∗∗	-64.02	-7.957∗∗∗	-73.909
**NSES Q2**	-3.18∗∗∗	-22.60	-2.51∗∗∗	-20.87	-2.39∗∗∗	-20.12	-2.371∗∗∗	-24.614
**NSES Q4**	1.48∗∗∗	10.52	1.17∗∗∗	9.84	1.13∗∗∗	9.59	1.452∗∗∗	15.077
**NSES Q5**	3.02∗∗∗	21.46	2.98∗∗∗	24.91	2.83∗∗∗	23.90	3.225∗∗∗	31.930
**Neighborhood density**			0.00∗∗∗	-19.61	0.00∗∗∗	-17.89	-0.000∗∗∗	-13.225
**Over 65 years**			-0.28∗∗∗	-67.05	-0.28∗∗∗	-68.46	-0.272∗∗∗	-73.471
**Spatial SES spillover θ**					176.11∗∗∗	17.54	113.447∗∗∗	9.246
**λ**							0.725∗∗∗	65.675

∗∗∗ p ​< ​0.001, ∗∗ ​< ​0.01, ∗ ​< ​0.05, ref NSES is Q3.

Model 3 shows that the coefficient for the spatially weighted NSES variable is significant and positive: neighborhoods adjacent to higher NSES neighborhoods have on average higher SAH, while neighborhoods adjacent to lower NSES neighborhoods are negatively affected. The GNS (Supplementary Table S1) model reveals a significant coefficient for *λ* (spatial error) but no significant term for ρ (spatial autcorrelation in the dependent variable). This indicates that the spatial autocorrelation coefficient for the dependent variable can be removed from the model, but that the spatial structures in the error term should be accounted for. We subsequently model this in the SPD-E model (Model 4) and find most coefficients unchanged compared to the GNS model, and similar in sign and significance to the SLX model, although the estimated impact of lagged NSES is lower in the model accounting for spatial autocorrelation of the error term. In the SAC model, where the spatial spillover effect of NSES is constrained to zero, the ρ term is significant. In all models, the direct association between NSES and SAH remained intact (p < 0.001). Albeit in the opposite direction, the same results were obtained for CD (Table 4), with the exception that for CD the estimate for spatial autocorrelation in the dependent variable was significant in all models.

**Table 4:** Chronic Diseases model estimates.

	Model 1: β	Model 1: t-value	Model 2: β	Model 2: t-value	Model 3: β	Model 3: t-value	Model 4 SPD-E: β	Model 4 SPD-E: t
**(Intercept)**	31.86∗∗∗	352.360	25.5097∗∗∗	259.393	25.58∗∗∗	256.490	25.855∗∗∗	203.750
**NSES Q1**	6.82∗∗∗	53.34	5.98∗∗∗	59.46	5.29∗∗∗	51.15	4.715∗∗∗	56.280
**NSES Q2**	2.79∗∗∗	21.88	2.11∗∗∗	21.77	1.99∗∗∗	20.95	1.771∗∗∗	23.689
**NSES Q4**	-1.33∗∗∗	-10.46	-1.02∗∗∗	-10.63	-0.98∗∗∗	-10.37	-1.106∗∗∗	-14.792
**NSES Q5**	-2.15∗∗∗	-16.85	-2.15∗∗∗	-22.36	-2.00∗∗∗	-21.16	-2.447∗∗∗	-31.110
**Neighborhood density**	–	–	0.00∗∗∗	19.44	0.00∗∗∗	17.38	0.000∗∗∗	20.063
**Over 65 years**	–	–	0.31∗∗∗	93.71	0.32∗∗∗	96.34	0.302∗∗∗	105.128
**Spatial SES spillover θ**	–	–	–	–	-178.14∗∗∗	-22.17	-5,6.389∗∗∗	-5.772
**λ**	–	–	–	–	–	–	0.765∗∗∗	76.424

∗∗∗ p ​< ​0.001, ∗∗ ​< ​0.01, ∗ ​< ​0.05, ref NSES is Q3.

### Policy scenario

Table 5 shows the change in the percentage of individuals with SAH or CD to illustrate the expected effect of a hypothesized policy scenario aimed at improving the NSES for the lowest NSES neighborhoods. Given the spatial spillovers identified in the preceding section, any increase in NSES will affect SAH both directly and indirectly through spillovers from neighboring regions. To account for these effects, the results were calculated using the predict.sarlm function in the spatialreg package. Following the terminology in Bivand (2002), we calculated in-sample (for the same regions but with new variables) predictions for the spatial Durbin error models. “TS” predictions were used for both the SAH and CD.

**Table 5:** Policy scenarios: Expected changes in self-assessed health and chronic diseases.

Improvement of lowest Quintile NSES to second lowest Quintile NSES				
	Self-assessed health - Original (sd)	Self-assessed health - Scenario (sd)	Chronic diseases - Original (sd)	Chronic diseases - Scenario (sd)
**NSES Q1**	68.7 (6.7)	75.1 (2.9)	38.7 (5.4)	34.7 (3.2)
**NSES Q2**	75.4 (4.4)	75.5 (2.2)	34.7 (3.8)	34.4 (2.5)
**NSES Q3**	78.6 (3.9)	78.5 (2.0)	31.9 (3.8)	31.9 (2.2)
**NSES Q4**	80.1 (3.7)	80.3 (2.1)	30.5 (4.0)	30.4 (2.4)
**NSES Q5**	81.6 (4.5)	81.9 (2.8)	29.7 (4.5)	29.4 (3.2)

The policy scenario investigated here involves lifting all the NSES for all the neighborhoods in the first quintile to the lower level of NSES in the second quintile. As a consequence, for two adjacent neighborhoods of which one is low-NSES and the other high-NSES, the overall NSES composition is enhanced. The results show that the expected increase in SAH for the neighborhoods in the lowest (current) quintile of NSES would be 6.4 percentage points. The second and third quintile neighborhoods do not benefit substantially, while the top two quintiles see moderate positive spillovers in this policy scenario. For CD, the largest prospective benefits are similarly expected in the lowest quintile. However, for CD the second quintile and top quintile also show small improvements. The third and fourth quintiles do not show much improvement.

## Discussion

In this paper we aimed to estimate population health inequalities, both within and between neighborhoods with similar socioeconomic status, and assessed the association of neighborhood characteristics and socioeconomic spillover effects from adjacent neighborhoods. We found substantial population health disparities in the percentage of individuals reporting to be in good or very good health and the percentage of individuals with one or more CD, both between neighborhoods with different and neighborhoods with similar socioeconomic characteristics. These differences were only partially explained by population density and the share of individuals over 65 years of age. Neighborhoods adjacent to higher SES neighborhoods showed a higher SAH and a lower prevalence of CD, adjusted for other explanatory variables. A hypothetical policy scenario targeting the lowest NSES group revealed substantial health gains resulting from both direct effects (for the lowest NSES group) and indirect effects (for all NSES groups) due to spatial spillover effects.

The substantial variation in subjective and objective population health measures (as well as lifestyle factors) between neighborhoods with similar socioeconomic characteristics presented in this paper, may potentially directly provide policy anchors for interventions that improve population health in disadvantaged neighborhoods without changing socioeconomic characteristics, which are notoriously more difficult to alter. Only a handful of studies have documented the differences in health outcomes between socioeconomically similar neighborhoods. Focusing on cost data from the Netherlands, de Boer et al. (2019) showed that healthcare costs of the most deprived NSES exhibited substantial variation, with some displaying health care costs well below the average costs of high NSES neighborhoods. In addition, Ferrer and Palmer (2004) observed considerable variability in self-rated health within socioeconomic strata. There was a resilient subgroup of lower SES people whose self-rated health remained excellent throughout life, while in a vulnerable group of low SES persons a rapid deterioration in health status as they reach middle age was observed. Clearly, more insight is needed using a priori designed studies to evaluate the potential of social determinant-related interventions to improve health outcomes and reduce health disparities within and across groups of neighborhoods with similar socioeconomic status.

In this study we showed the potential role of population density and the effect of spatial spillovers in the variation in subjective and objective health within and across neighborhoods with similar socioeconomic characteristics. Neighborhoods adjacent to higher NSES neighborhoods showed on average higher SAH and lower prevalence of CD. This seems especially true for neighborhoods with the lowest NSES scores, as the present results showed that this group was most affected by regional SES spillover effects. The underlying motivation for spatial thinking is grounded in Tobler's first law of geography “everything is related to everything else, but near things are more related than distant things”(Miller, 2004). These, in turn, convey a spatial perspective that the characteristics of a neighborhood are not merely shaped by a particular bounded location, but are also shaped by the characteristics of its surrounding locations. While the mechanisms behind the observed effect of socioeconomic spillovers on health needs further study, it may already provide interesting leads to policy design aimed at improving population health outcomes of deprived neighborhoods. For example, the present results may urge for designing more socio-economically mixed regions of neighborhoods, with the idea that poor neighborhoods could benefit from the presence of, and interaction with more affluent neighborhoods (Galster & Friedrichs, 2015).

Additionally, this study presented an evidence base for the potential of improving population health when targeting fundamental causes of health disparities in the most deprived neighborhoods. Small changes in NSES might already have substantial improvements in SAH and CD. To further illustrate the potential of such interventions, de Boer et al. (2019) calculated potential health care cost savings by reducing health differences between neighborhoods with comparable income and education levels. If each neighborhood had the same health care expenditures as the average neighborhood with the same socioeconomic characteristics, health care costs could still be reduced by 2.4% of overall health care expenditures. As shown in the present study, not only the target group may experience substantial health gains through such intervention, also the total population might benefit from it, due to the potential of spatial spillover effects. Previously this was also concluded by Benjamin-Chungough et al. (2017), who stated that interventions may benefit not only direct recipients but also those who did not receive the intervention but are connected to the recipients.

A further and crucial implication of the within group health disparities is that inequities in health apply to everyone. Therefore, social action should deal with the entire gradient, and all of society, not only with those at the bottom. Questions about the effect of universal versus targeted prevention strategies on population health and health inequalities, and the role that fundamental causes play in population health, are critical to the articulation of effective public health planning strategies. Ideally, a universal course in increasing SES potential must be chosen, according to the theory of the proportionate universalism of the British epidemiologist Michael Marmot (Marmot & Bell, 2012; Marmot & Health, 2007). That means that prevention policy, while targeting all citizens, is being complemented by support for certain target groups. The intensity of this targeted support is determined by the degree of vulnerability of the target group. Additionally, positioning health equity as a key performance indicator in all social and economic policy has the potential to drive significant reductions in health inequities.

While we used rich, whole-population data, on both subjective and objectively measured health outcomes, this study is not without limitations. First of all, because our study focused on the neighborhood level, the findings may not be fully transferable to policies and interventions aimed at the individual level. Indeed, due to the reliance on neighborhood-level data we were not able to adjust the data for age and sex. Second, because of the unique characteristics of the Dutch health care system and Dutch society (e.g., the country's rather egalitarian socioeconomic structure), the outcomes of this research may not be fully generalizable to other countries. However, because of the scope of this research, we believe that the findings provide valuable insights, as would similar investigations in other countries. Third, because we used a cross-sectional approach, the associations presented are not necessarily indicative of causal relations.

## Conclusion

Population health differs substantially among neighborhoods with similar socioeconomic characteristics, which can partially be explained by a spatial socioeconomic spillover effect. The mechanisms behind these socioeconomic spillovers need further study, and this study has shown that spatial spillovers may provide interesting leads to policy design aimed at improving population health outcomes of deprived neighborhoods.

## Declaration of competing interest

The authors declare that they have no competing interests.

## List of abbreviations

BMI: body mass index
CD: chronic diseases
GAM: generalized additive models
NSES: neighborhood socioeconomic status
SES: socioeconomic status
SAH: self-assessed health

## Availability of data and materials

The data may be requested from The National Institute for Public Health and Statistics Netherlands directly.
