# Supplementary material for: The health potential of neighborhoods: A population-wide study in the Netherlands
Source: SSM Popul Health. 2021 Jul 7;15:100867. doi: 10.1016/j.ssmph.2021.100867 (PMC8327128; doi:10.1016/j.ssmph.2021.100867)
Supplement: Multimedia component 1 [file mmc1.docx]

# **Supplementary material**

**The Health Potential of Neighborhoods: A population-wide study in the Netherlands**

Dekker LH ^1,2^, Rijnks RH ^3^, Mierau, JO ^2,4^

1. University Medical Center Groningen, Department of Nephrology, Hanzeplein 1, 9713 GZ Groningen, the Netherlands

2. Aletta Jacobs School of Public Health, Landleven 1, 9747 AD Groningen, the Netherlands

3. University College Cork, Cork University Business School, West Wing, Main Quadrangle, T12 K8AF, Ireland

4. University of Groningen, Faculty of Economics and Business, Nettelbosje 2, 9747 AE Groningen, the Netherlands


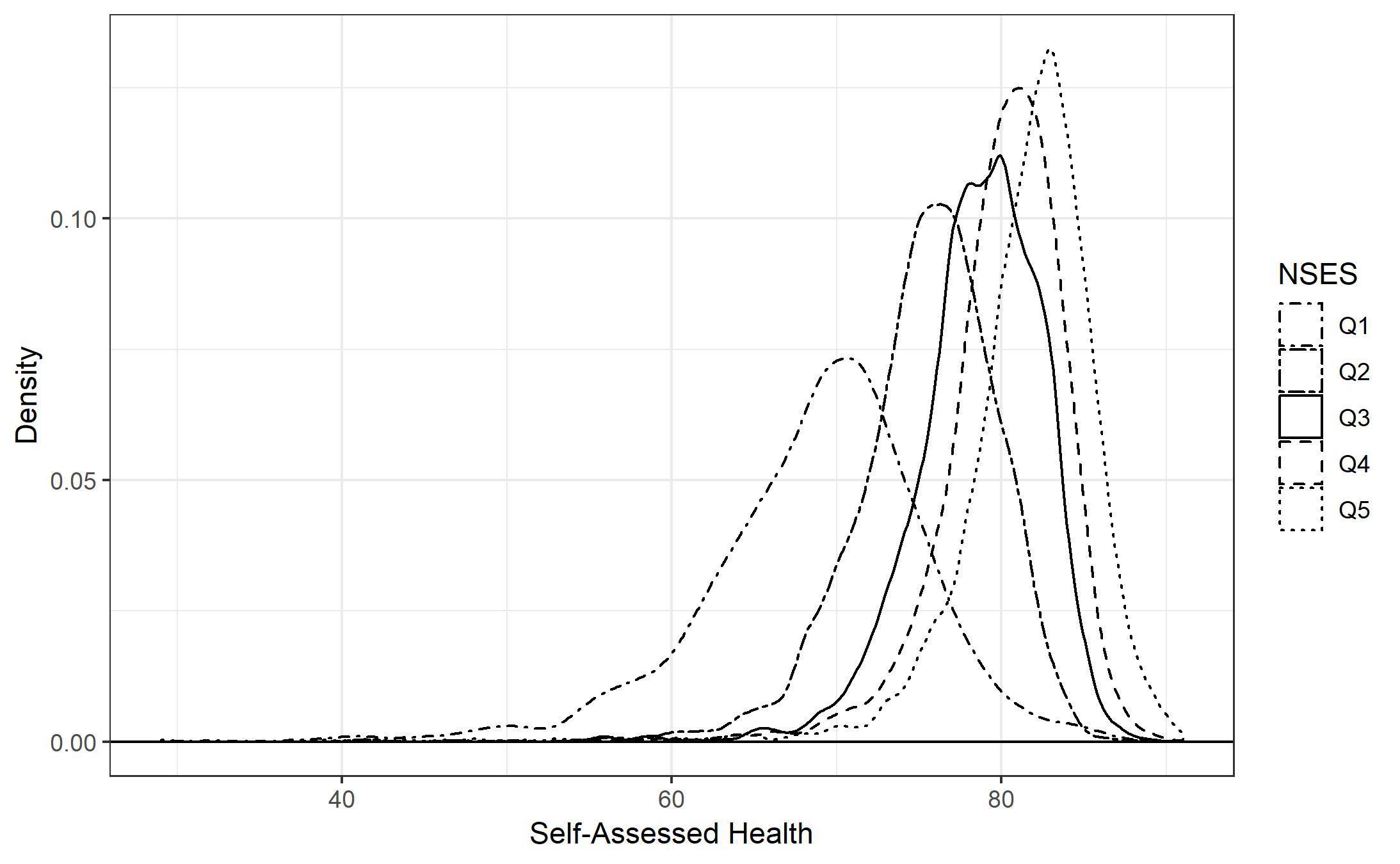


**Figure S1a.** Self assessed health according to quintiles of neighborhood SES


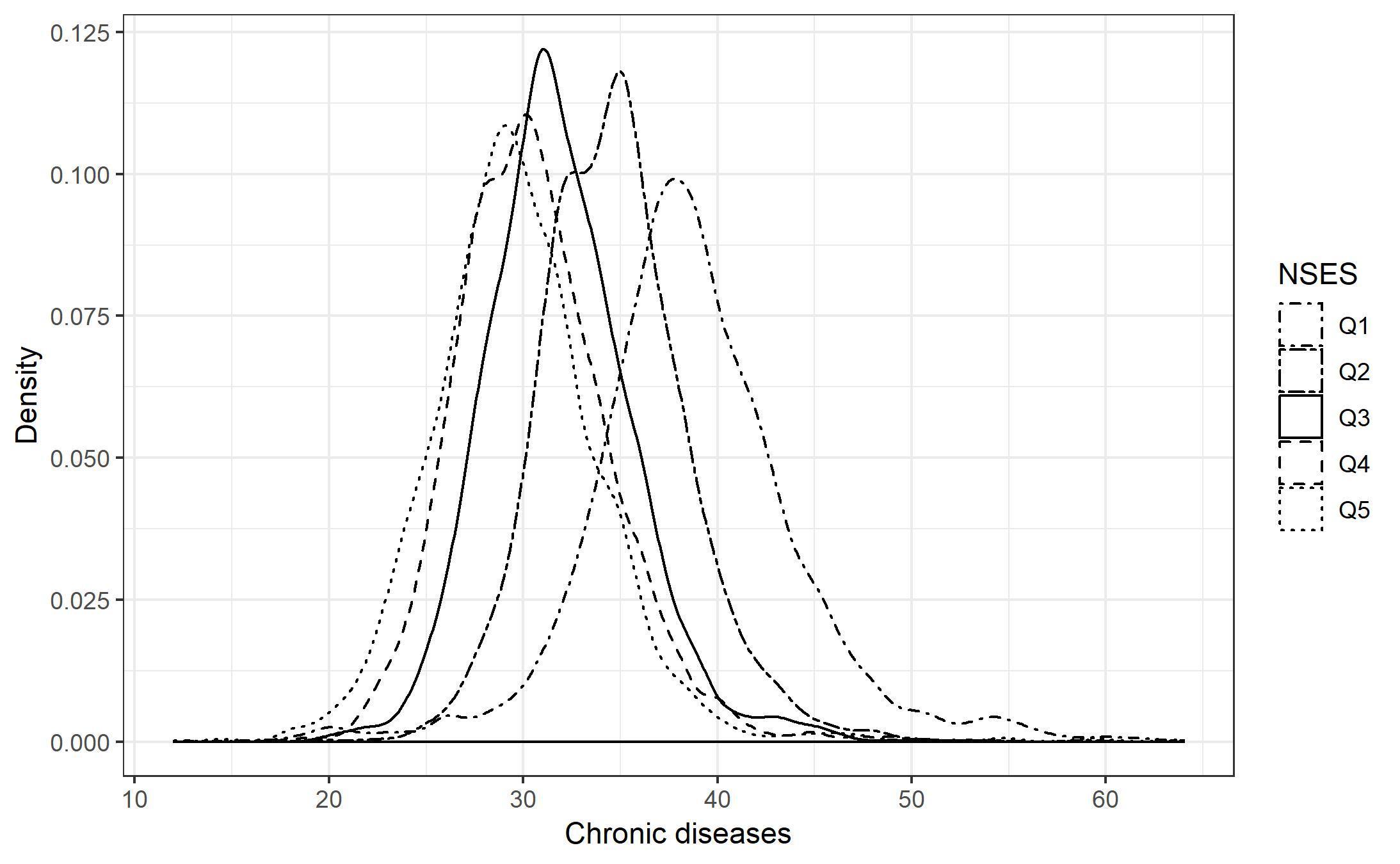


**Figure S1b**. Chronic disease according to quintiles of neighborhood SES

**Table S1. Variation of self-assessed health, chronic diseases and lifestyle measures within and between quintiles of neighboorhood socioeconomic status**

|  | **Self assessed health** | **Chronic diseases** | **Sports** | **Alcohol recommendation** | **Overweight** | **Non-**  **smokers** |
| --- | --- | --- | --- | --- | --- | --- |
| **Between** | 5.12 | 3.65 | 4.30 | 3.99 | 2.20 | 3.80 |
| **Within** | 4.66 | 4.29 | 7.02 | 6.37 | 5.28 | 3.75 |
| **Overall** | 6.62 | 5.43 | 8.05 | 7.38 | 5.70 | 5.09 |

**Table 2. SAH Spatial Estimates**

|  | **Model 1 GNS: β** | **Model 1 GNS: t** | **Model 2 SAC: β** | **Model 2 SAC: t** |
| --- | --- | --- | --- | --- |
| **rho** | 0.003 | 0.028 | 0.170*** | 8.913 |
| **(Intercept)** | 83.549*** | 39.077 | 70.797*** | 47.982 |
| **NSES Q1** | -7.957*** | -73.875 | -7.991*** | -73.639 |
| **NSES Q2** | -2.371*** | -24.608 | -2.354*** | -24.122 |
| **NSES Q4** | 1.452*** | 15.070 | 1.425*** | 14.607 |
| **NSES Q5** | 3.225*** | 31.907 | 3.174*** | 31.243 |
| **Neighborhood density** | -0.000*** | -13.219 | -0.000*** | -13.787 |
| **Over 65 years** | -0.272*** | -73.117 | -0.273*** | -73.353 |
| **Spatial SES spillover** | 111.947*** | 6.253 | - | - |
| **lambda** | 0.723*** | 41.862 | 0.639*** | 36.145 |

*** p < 0.001, ** < 0.01, * < 0.05, ref NSES is Q3

**Table 3. Chronic Spatial Estimates**

|  | **Model 1 GNS: β** | **Model 1 GNS: t** | **Model 2 SAC: β** | **Model 2 SAC: t** |
| --- | --- | --- | --- | --- |
| **rho** | 0.,101*** | 4.,137 | 0.,019*** | 7.,804 |
| **(Intercept)** | 22.,507*** | 27.,365 | 21.,029*** | 33.,605 |
| **NSES Q1** | 4.,741*** | 56.,301 | 4.,751*** | 56.,326 |
| **NSES Q2** | 1.,787*** | 23.,699 | 1.,784*** | 23.,601 |
| **NSES Q4** | -1.,112*** | -14.,745 | -1.,107*** | -14.,648 |
| **NSES Q5** | -2.,448*** | -30.,983 | -2.,437*** | -30.,857 |
| **Neighborhood density** | 0.,000*** | 20.,047 | 0.,000*** | 20.,317 |
| **Over 65 years** | 0.,304*** | 104.,739 | 0.,305*** | 104.,999 |
| **Spatial SES spillover** | -27.,973*** | -2.,232 | - | - |
| **lambda** | 0.,716*** | 42.,544 | 0.,695*** | 44.,500 |

*** p < 0.001, ** < 0.01, * < 0.05, ref NSES is Q3
